# The trend in primary health care preference in China: a cohort study of 12,508 residents from 2012 to 2018

**DOI:** 10.1186/s12913-021-06790-w

**Published:** 2021-08-03

**Authors:** Guangsheng Wan, Xiaolin Wei, Hui Yin, Zhiwang Qian, Tingting Wang, Lina Wang

**Affiliations:** 1 School of Nursing and Health Management, Shanghai University of Medicine & Health Sciences, Shanghai, 201318 China; 2 Dalla Lana School of Public Health, University of Toronto, Toronto, ON M5T3M6 Canada; 3 School of Health Management, Harbin Medical University, Harbin, 150081 Heilongjiang China; 4 Foreign Language Faculty, Shanghai University of Medicine & Health Sciences, Shanghai, 201318 China

**Keywords:** China, Primary health care, Determinants of preference, Trend of preference, Health system

## Abstract

**Background:**

Residents’ preference for primary health care (PHC) determined their utilization of PHC. This study aimed to assess the determinants of PHC service preference among the residents and the trend in PHC service preference over time in China.

**Methods:**

We employed the nationally representative longitudinal data from 2012 to 2018 based on the China Family Panel Studies. The analysis framework was guided by the Andersen model of health service utilization. We included a total of 12,508 individuals who have been successfully followed up in the surveys of 2012, 2014, 2016, and 2018 without any missing data. Logistic regressions were performed to analyze potential predictors of PHC preference behavior.

**Results:**

The results indicated that individuals’ socio-economic circumstances and their health status factors were statistically significant determinants of PHC preference. Notably, over time, the residents’ likelihood of choosing PHC service represented a decreasing trend. Compare to 2012, the likelihood of PHC service preference decreased by 18.6% (OR, 0.814; 95% CI, 0.764–0.867) in 2014, 30.0% (OR, 0.700; 95% CI, 0.657–0.745) in 2016, and 34.9% (OR, 0.651; 95% CI, 0.611–0.694) in 2018. The decrease was significantly associated with the changes in residents’ health status.

**Conclusions:**

The residents’ likelihood of choosing PHC service represented a decreasing trend, which was contrary to the objective of China’s National Health Reform in 2009. We recommend that policymakers adjust the primary service items in PHC facilities and strengthen the coordination of service between PHC institutions and higher-level hospitals.

**Supplementary Information:**

The online version contains supplementary material available at 10.1186/s12913-021-06790-w.

## Background

During the past decade of healthcare reform, China has made great efforts to improve the Primary Health Care (PHC) system [1], especially to strengthen the construction of PHC delivery system. In China, the PHC institutions consist of community healthcare centers/stations in urban areas, township hospitals, and village clinics in rural areas [2]. They provide general disease diagnosis, treatment services, and essential public health services [3, 4]. After the National Health Reform in 2009, PHC institutions witnessed unprecedented investment from the government [5], reaching to tenfold of the funds in 2018 as compared to that in 2008 [1]. Meanwhile, a series of policies, such as discriminatory compensation in medical insurance and dual referral in regional medical association [6], were implemented to encourage the residents to use the PHC services. According to *China Health Statistics Yearbook 2019*, the number of PHC institutions in mainland China was 943,639 in 2018, with an increase of 41,930 since 2010. Those facilities improved the access to primary health care to residents [5]. However, PHC utilization is comparatively lower than that of non-PHC institutions in China, and the doctors’ outpatient volume in higher-tier hospitals is six to ten times more than those in PHC institutions [7]. Patients’ preference for different types of medical institutions determined the PHC utilization in China [8]. Studies on the PHC preference can help optimize the PHC service delivery system, but it was far from sufficient.

Previous studies about PHC preference mainly concentrated on the determinants of choosing PHC services and limited in research data. For example, characteristics of care provider, service mode, cost or cost-sharing policy, individuals’ characteristics, and their experiences with PHC were the key factors impacting the preference for PHC [2, 8–11]. The provision and utilization of PHC in China varied from region to region due to large differences in socioeconomic factors and health policies [12, 13]. Most of the studies about PHC preference in China were based on cross-sectional surveys in limited geographic areas [2, 10, 14]. Thus, they cannot be extrapolated to the general population in China [11]. Besides, the preference for PHC is not immutable. We found that, over time, many residents changed their preferences. Most studies did not discuss the preference shifting behavior or investigate factors related to people who changed their preference for health care institutions. The lack of studies on the change of PHC preference is not conducive to the construction of PHC service delivery system. It is crucial for policymakers to understand the determinants of PHC preference shifting behavior.

This study aims to assess the determinants of PHC service preference among residents in China and assess the temporal changes in preference over the study period. We used the Andersen model of health service utilization to guide the analysis framework. The nationally representative longitudinal data from 2012 to 2018 was used to examine the relationship between predictors and preference behavior. At first, we analyzed the determinants of PHC preference and found that the likelihood of choosing PHC decreased over time. Then, we discussed the decreasing trend from the perspective of preference shifting. Our study has two contributions. Firstly, it can contribute to the existing literature by providing new findings about determinants of PHC preference in China, and used nationwide longitudinal data to demonstrate the new trend. Secondly, the results provide a reference for health sectors to focus on the cause of PHC preference trend and provide a reference to adjust their health policies. Policymakers need to pay attention to the changing of residents’ PHC needs and adjust the service items in time.

## Data and methods

### Data

Data were obtained from China Family Panel Studies (CFPS), maintained by the Institute of Social Science Survey (ISSS) at the Peking University. This survey was launched in 2008, and the first wave was conducted in 2010 by ISSS, which conducts the tracking survey every two years. The multi-stage probability sampling design with an implicit stratification method was used in the baseline survey. First, 25 provincial-level administrative regions (excluding Hong Kong, Macao, Taiwan, Tibet, Xinjiang, Qinghai, Inner Mongolia, Ningxia, and Hainan) in mainland China were included, and the primary sampling units were produced according to socioeconomic status. Second, within each primary sampling unit, villages or communities were selected with the systematic probability proportional to size sampling method. Finally, 28 to 42 households were selected in each village or community by cyclic equidistant sampling method, and face-to-face interviews were conducted with adults. Details of the design of CFPS can be found on their website: http://www.isss.pku.edu.cn/cfps.

**Fig. 1:**
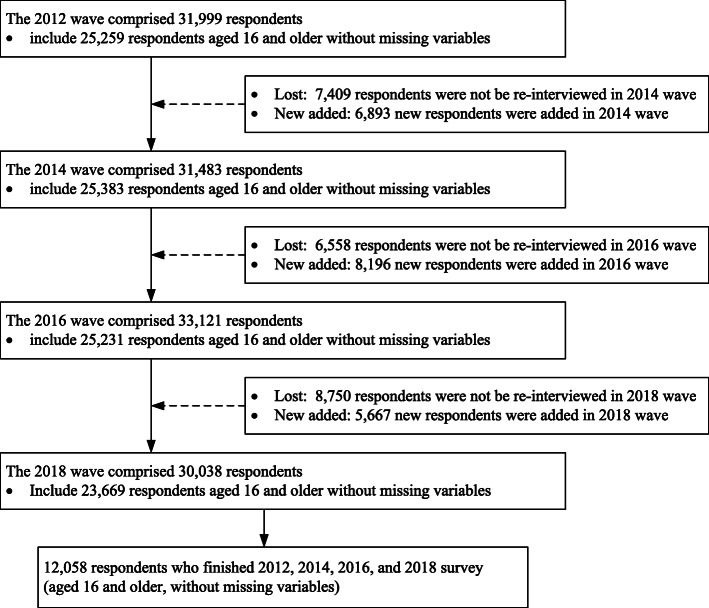
Flow chart of the selection of respondents

The CFPS represents 94.5% of the mainland population in China. In the baseline year of 2010, 14,960 households and 42,590 individuals in those households were interviewed. The individuals were surveyed every two years, and added new samples to make up for lost samples. Our study used the datasets of 2012, 2014, 2106, and 2018 waves and focused on the individuals aged 16 years or older who were asked to provide complete information on their primary health care preference. The number of original respondents in each wave was showed in Fig. 1. First, we followed each wave of the survey, and obtained respondents with complete information. There were 25,259 respondents in the 2012 survey, 25,383 respondents in the 2014 survey, 25,231 respondents in the 2016 survey, and 23,669 respondents in the 2018 survey. Second, we obtained the 16,718 respondents who completed all the four waves of survey. Of them, we further excluded respondents who had missing data (4660, 27.9%) and included a total of 12,058 of them without missing data. Thus, our analysis sample is 12,058 individuals, with each having four rounds of interview (i.e., a total of 12,058 × 4 = 48,232 respondents).

### Measures

#### Outcome variable: PHC preference and preference change

The dependent variable in this study was whether the respondents usually chose PHC services or not. This variable was evaluated by the question, “Where would you usually go to see a doctor?” The respondents chose their answers from the following places, including non-PHC institutions (including general hospitals and specialty hospitals) and PHC institutions (including community healthcare center/township hospital, community healthcare station/village clinic, and clinic). The dependent variable is the preference for PHC services (1 = choosing PHC institutions, and 0 = choosing non-PHC institutions). In analyzing the change of preference, the dependent variable was the change of preference for medical institution compared with the last wave of survey. It was classified into three categories: from PHC to non-PHC, from non-PHC to PHC, and non-shifting.

#### Explanatory variables

The Andersen model of health service utilization was used to guide our analysis of PHC services preference determinants. The Andersen model was widely used in the literature [15–19], and advances three sets of factors to account for health services utilization. In this study, we focused on PHC service preference. We hypothesize that an individual’s preference for PHC determines his utilization of PHC services based on three sets of factors, including predisposing, enabling, and needs-based factors [20, 21]. The three sets of explanatory variables used in our work were listed as follows.

First, predisposing factors included gender, age, education level, ethnicity, marital status, and household size. Educational level was divided into three categories: junior high school and below, high school or secondary, and university/college or above. The ethnicity of respondents was coded as Han ethnicity or other minority ethnicities. Marital status was divided into married and unmarried groups (including single, unmarried cohabitation, divorced, and death of a spouse). According to the number of household members, household was classified into three categories: 1–2 persons, 3–4 persons, and five or above.

Second, enabling factors included household income, employment status, Hukou, social basic medical insurance status, geographical region, and living area. Household income in the past 12 months was classified into six groups: below 30 thousand RMB, 30–50 thousand RMB, 50–70 thousand RMB, 70–90 thousand RMB, 90 thousand RMB and above. The employment status of respondents was divided into the current working group and not currently working group. According to China’s household registration system, Hukou status was classified into agricultural and non-agricultural groups. Social basic medical insurance provides support for individuals to use PHC services, and there are three main programs in China, including Urban Employee Basic Medical Insurance (UEBMI), New Cooperative Medical Scheme (NCMS), and Urban Resident Basic Medical Insurance (URBMI). Besides, a small number of individuals hold the Free Medical Service (FMS), which has been gradually phased out since 1998. Therefore, the social basic medical insurance was classified into UEBMI, NCMS, URBMI, and FMS. According to China’s administrative division, the provinces were classified into eastern, central, and western regions. The living area was also divided into urban areas and rural areas.

Third, needs-based factors included self-rate health status, whether having any doctor-diagnosed chronic disease or not during the past six months, and whether been hospitalized due to illness in the past year. Self-rate health status was classified into three categories: poor, fair, and good. Chronic disease and hospitalization were both assigned as 0–1 dummy variables. Meanwhile, the changing of health status between different survey waves was also coded, and it was classified into three categories: become worse, become better, and no changing.

Finally, year dummy variables were also included to verify the time effect in PHC services preference.

### Statistical analysis

In analyzing the determinants of PHC preference, whether an individual usually chose PHC services or not was the dependent variable. The independent variables were the three sets of factors suggested by the Andersen model and the year dummy variables. Logistic regression and Chi-square tests were used to perform the empirical analysis. First, descriptive statistics and chi-square tests of independent variables were performed to examine whether the differences between PHC preference and non-PHC preference in terms of each potential predictor was statistically significant (Table 1). Other health related information and preference shifting information were also described in Table 2, Table 3, and Table 4. Second, univariate logistic regression was performed to verify each predictor’s crude relationship with the dependent variable (Model 1 in Table 5). Third, based on the univariate analysis results, multivariate logistic regression was performed to analyze potential predictors of PHC preference behavior (Model 2 in Table 5). We used the backward stepwise method to calculate the multivariate logistic regression. A significance level of 0.1 was set to let the independent variables enter the multivariate model. Fourth, multinomial logistic regressions were also performed to examine the relationship between the individuals’ preference shifting behavior and their health status changes (Table 6). Finally, we also provided the descriptive information and performed the logistic regression analysis by using pooled four rounds of data to check the robustness of the results (Table A1, Table A2 in Appendix A). Logistic regression models were also performed when dividing the sample into sub-groups by geographical variables (Table A3 in Appendix A). All the above logistic regression results, except for multinomial logistic regression, were reported as odds ratios (ORs) and 95% confidence intervals (CIs). Stata 14.0 was used to perform the data analysis.

**Table 1:** Descriptive information on PHC preference under Andersen Model

Factors	2012 (*n* = 12,058)		2014 (*n* = 12,058)		2016 (*n* = 12,058)		2018 (*n* = 12,058)	
	PHC N (%)	Non-PHC N (%)	PHC N (%)	Non-PHC N (%)	PHC N (%)	Non-PHC N (%)	PHC N (%)	Non-PHC N (%)
N	8758 (72.63)	3300 (27.37)	8324 (69.03)	3734 (30.97)	7916 (65.65)	4142 (34.35)	7569 (62.77)	4489 (37.23)
**Predisposing factors**								
Gender								
Female	4532 (73.62)	1709 (27.38)	4292 (68.85)	1942 (31.15)	4072 (65.33)	2161 (34.67)	3922 (62.89)	2314 (37.11)
Male	4226 (72.65)	1591 (27.35)	4032 (69.23)	1792 (30.77)	3844 (65.99)	1981 (34.01)	3647 (62.64)	2175 (37.36)
	*χ*^2^=0.002; *P* = 0.968		*χ*^2^=0.206; *P* = 0.650		*χ*^2^=0.580; *P* = 0.444		*χ*^2^=0.081; *P* = 0.776	
Age								
16 ~ 25	530 (76.59)	162 (23.41)	261 (72.10)	101 (27.90)	95 (66.43)	48 (33.57)	38 (63.33)	22 (36.67)
26 ~ 35	1198 (72.92)	445 (27.08)	1074 (67.12)	526 (32.88)	990 (66.40)	501 (33.60)	795 (65.70)	415 (34.30)
36 ~ 45	2236 (74.16)	779 (25.84)	1081 (68.43)	831 (31.57)	1478 (65.56)	781 (34.44)	1275 (63.50)	733 (36.50)
46 ~ 55	2187 (72.15)	844 (27.85)	2277 (70.74)	942 (29.26)	2337 (67.02)	1150 (32.98)	2327 (64.93)	1257 (35.07)
56 ~ 65	1904 (72.42)	725 (27.58)	1979 (69.88)	853 (30.12)	1828 (65.40)	967 (34.60)	1717 (61.96)	1054 (38.04)
≥ 66	703 (67.08)	345 (32.92)	932 (65.96)	481 (34.04)	1179 (62.91)	695 (37.09)	1417 (58.43)	1008 (41.57)
	*χ*^2^=25.728; *P* = 0.000		*χ*^2^=16.334; *P* = 0.006		*χ*^2^=9.619; *P* = 0.087		*χ*^2^=32.343; *P* = 0.000	
Education								
Junior high school and below	7544 (77.20)	2228 (22.80)	7222 (73.93)	2547 (26.07)	6908 (70.79)	2851 (29.21)	6493 (67.52)	3124 (32.48)
High school or secondary	932 (60.76)	602 (39.24)	861 (56.53)	662 (43.47)	785 (51.78)	731 (48.22)	838 (53.27)	735 (46.73)
University or college and above	282 (37.50)	470 (62.50)	241 (31.46)	525 (68.54)	223 (28.48)	560 (71.52)	238 (27.42)	630 (72.58)
	*χ*^2^=678.361; *P* = 0.000		*χ*^2^=726.595; *P* = 0.000		*χ*^2^=723.167; *P* = 0.000		*χ*^2^=617.557; *P* = 0.000	
Ethnicity								
Han	8158 (72.43)	3105 (27.57)	7743 (68.75)	3520 (31.25)	7354 (65.29)	3909 (34.71)	7053 (62.62)	4210 (37.38)
Minority	600 (75.47)	195 (24.53)	581 (73.08)	214 (26.92)	562 (70.69)	233 (29.31)	516 (64.91)	279 (35.09)
	*χ*^2^=3.452; *P* = 0.063		*χ*^2^=6.526; *P* = 0.011		*χ*^2^=9.596; *P* = 0.002		*χ*^2^=1.659; *P* = 0.198	
Marital status								
Unmarried	705 (69.25)	313 (30.75)	687 (66.89)	340 (33.11)	678 (65.26)	361 (34.74)	704 (60.07)	468 (39.93)
Married	8053 (72.94)	2987 (27.06)	7637 (69.23)	3394 (30.77)	7238 (65.69)	3781 (34.31)	6865 (63.06)	4021 (36.94)
	*χ*^2^=6.386; *P* = 0.012		*χ*^2^=2.403; *P* = 0.121		*χ*^2^=0.078; *P* = 0.779		*χ*^2^=4.060; *P* = 0.044	
Household size								
1 ~ 2 person	1223 (65.61)	641 (34.39)	1306 (61.49)	818 (38.51)	1363 (58.75)	957 (41.25)	1544 (55.34)	1246 (44.66)
3–4 person	3534 (69.61)	1543 (30.39)	3161 (65.27)	1682 (34.73)	2890 (63.64)	1651 (36.36)	2651 (60.87)	1704 (39.13)
≥ 5 person	4001 (78.19)	1116 (21.81)	3857 (75.76)	1234 (24.24)	3663 (70.48)	1534 (29.52)	3374 (68.67)	1539 (31.33)
	*χ*^2^=149.104; *P* = 0.000		*χ*^2^=196.458; *P* = 0.000		*χ*^2^=110.925; *P* = 0.000		*χ*^2^=145.916; *P* = 0.000	
**Enabling factors**								
Household income								
< 30 thousand	3941 (78.38)	1087 (21.62)	3749 (76.32)	1163 (23.68)	3667 (75.42)	1195 (24.58)	2987 (71.10)	1214 (28.90)
30 ~ 50 thousand	1828 (71.32)	735 (28.68)	1981 (70.10)	845 (29.90)	1594 (66.86)	790 (33.14)	1730 (68.43)	798 (31.57)
50 ~ 70 thousand	1172 (69.93)	504 (30.07)	941 (62.65)	561 (37.35)	947 (61.10)	603 (38.90)	941 (59.37)	644 (40.63)
70 ~ 90 thousand	691 (68.48)	318 (31.52)	550(58.70)	387 (41.30)	528 (54.72)	437 (45.28)	511 (56.22)	398 (43.78)
≥ 90 thousand	1126 (69.19)	656 (31.81)	1103 (58.64)	778 (41.36)	1180 (51.37)	1117 (48.63)	1400 (49.38)	1435 (50.62)
	*χ*^2^=180.677; *P* = 0.000		*χ*^2^=294.129; *P* = 0.000		*χ*^2^=480.505; *P* = 0.000		*χ*^2^=401.478; *P* = 0.000	
Employment status								
Not currently working	3341 (71.22)	1350 (28.78)	1327 (54.90)	1090 (45.10)	1334 (50.90)	1287 (49.10)	1312 (46.67)	1499 (53.33)
Currently working	5417 (73.53)	1950 (26.47)	6997 (72.58)	2644 (27.42)	6582 (69.75)	2855 (30.25)	6257 (67.67)	2990 (32.33)
	*χ*^2^=7.688; *P* = 0.006		*χ*^2^=282.340; *P* = 0.000		*χ*^2^=323.213; *P* = 0.000		*χ*^2^=406.473; *P* = 0.000	
Hukou								
Agricultural	7538 (81.22)	1743 (18.78)	7206 (78.06)	2025 (31.94)	6875 (74.80)	2316 (25.20)	6546 (71.31)	2633 (28.69)
Non-agricultural	1220 (43.93)	1557 (56.07)	1118 (39.55)	1709 (60.45)	1041 (36.31)	1826 (63.69)	1023 (35.53)	1856 (64.47)
	*χ*^2^=1.5 × 10^3^; *P* = 0.000		*χ*^2^=1.5 × 10^3^; *P* = 0.000		*χ*^2^=1.4 × 10^3^; *P* = 0.000		*χ*^2^=1.2 × 10^3^; *P* = 0.000	
Social basic medical insurance status								
UEBMI	606 (40.00)	909 (60.00)	544 (33.54)	1078 (66.46)	514 (30.50)	1171 (69.50)	531 (30.62)	1203 (69.38)
URBMI	337 (47.20)	377 (52.80)	370 (44.31)	465 (55.69)	341 (41.38)	483 (58.62)	332 (35.97)	591 (64.03)
NCMS	7679 (81.29)	1767 (18.71)	7305 (78.59)	1990 (21.41)	6998 (75.17)	2311 (24.83)	6632 (72.35)	2534 (27.65)
FMS	136 (35.51)	247 (64.49)	105 (34.31)	201 (65.69)	63 (26.25)	177 (73.75)	74 (31.49)	161 (65.81)
	*χ*^2^=1.7 × 10^3^; *P* = 0.000		*χ*^2^=1.8 × 10^3^; *P* = 0.000		*χ*^2^=1.7 × 10^3^; *P* = 0.000		*χ*^2^=1.5 × 10^3^; *P* = 0.000	
Geographic region								
East	3333 (68.41)	1539 (31.59)	3232 (66.19)	1651 (33.81)	3042 (61.97)	1867 (38.03)	2886 (58.41)	2055 (41.59)
Central	2728 (72.75)	1022 (27.25)	2555 (68.32)	1185 (31.68)	2416 (64.89)	1307 (35.11)	2337 (63.11)	1366 (36.89)
West	2697 (78.49)	739 (21.51)	2537 (73.86)	898 (26.14)	2458 (71.75)	968 (28.25)	2346 (68.72)	1068 (31.28)
	*χ*^2^=103.54; *P* = 0.000		*χ*^2^=56.776; *P* = 0.000		*χ*^2^=86.905; *P* = 0.000		*χ*^2^=92.060; *P* = 0.000	
Living area								
Rural	5886 (82.03)	1289 (17.97)	5444 (79.27)	1424 (20.73)	5170 (77.21)	1526 (22.79)	4740 (72.79)	1772 (27.21)
Urban	2872 (58.82)	2011 (41.18)	2880 (55.49)	2310 (44.51)	2746 (51.21)	2616 (48.79)	2829 (51.01)	2717 (48.99)
	*χ*^2^=788.016; *P* = 0.000		*χ*^2^=781.630; *P* = 0.000		*χ*^2^=892.448; *P* = 0.000		*χ*^2^=607.933; *P* = 0.000	
**Needs-based factors**								
Health status								
Good	5488 (75.20)	1810 (24.80)	5756 (71.17)	2332 (28.83)	5025 (67.84)	2382 (32.16)	5061 (65.12)	2711 (34.88)
Fair	1732 (71.51)	690 (28.49)	1295 (68.34)	600 (31.66)	1620 (64.70)	884 (35.30)	1159 (62.89)	684 (37.11)
Poor	1538 (65.78)	800 (34.22)	1273 (61.35)	802 (38.65)	1271 (59.20)	876 (40.80)	1349 (55.22)	1094 (44.78)
	*χ*^2^=80.896; *P* = 0.000		*χ*^2^=74.966; *P* = 0.000		*χ*^2^=56.402; *P* = 0.000		*χ*^2^=77.959; *P* = 0.000	
Chronic disease								
No	7720 (74.80)	2601 (25.20)	6989 (72.40)	2664 (27.60)	6591 (68.73)	2999 (31.27)	6239 (65.97)	3219 (34.03)
Yes	1038 (59.76)	699 (40.24)	1335 (55.51)	1070 (44.49)	1325 (53.69)	1143 (46.31)	1330 (51.15)	1270 (48.85)
	*χ*^2^=169.027; *P* = 0.000		*χ*^2^=257.016; *P* = 0.000		*χ*^2^=196.905; *P* = 0.000		*χ*^2^=191.451; *P* = 0.000	
Hospitalization								
No	8058 (73.72)	2872 (26.28)	7666 (72.12)	2963 (27.88)	7181 (68.82)	3253 (31.18)	6745 (66.66)	3373 (33.34)
Yes	700 (62.06)	428 (37.94)	658 (46.05)	771 (53.95)	735 (45.26)	889 (54.74)	824 (42.47)	1116 (57.53)
	*χ*^2^=70.017; *P* = 0.000		*χ*^2^=400.698; *P* = 0.000		*χ*^2^=346.028; *P* = 0.000		*χ*^2^=407.591; *P* = 0.000

**Table 2:** Information on chronic and hospitalization in different years

Variable		Year N (%)				
		2012	2014	2016	2018	
Hospitalization		12,058	12,058	12,058	12,058	*χ*^2^=261.024 *p* = 0.000
	No	10,930 (90.65)	10,629 (88.15)	10,434 (86.53)	10,118 (83.91)	
	Yes	1128 (9.35)	1429 (11.85)	1624 (13.47)	1940 (16.09)	
Chronic disease		12,058	12,058	12,058	12,058	*χ*^2^=239.524 *p* = 0.000
	No	10,321 (85.59)	9653 (80.05)	9590 (79.53)	9458 (78.44)	
	Yes	1373 (14.41)	2405 (19.95)	2468 (20.47)	2600 (21.56)

**Table 3:** Information on hospitalization with who had chronic disease in different years

Year		Hospitalization N (%)		Total N (***N*** = 12,058)	***χ***^**2**^	***P***
		No	Yes			
2012	Chronic disease				378.710	0.000
	No	9574 (92.76)	747 (7.24)	10,321		
	Yes	1356 (78.07)	381 (21.93)	1737		
2014	Chronic disease				744.570	0.000
	No	8896 (92.16)	757 (7.84)	9653		
	Yes	1733 (72.06)	672 (27.94)	2405		
2016	Chronic disease				947.673	0.000
	No	8763 (91.39)	826 (8.61)	9590		
	Yes	1670 (67.67)	798 (32.33)	2468		
2018	Chronic disease				943.553	0.000
	No	8466 (89.30)	1012 (10.70)	9458		
	Yes	1672 (64.31)	928 (35.69)	2600

**Table 4:** Information on preference shift and health status change in different years

Variable		Year N (%)		
		2012 → 2014	2014 → 2016	2016 → 2018
PHC preference shift		12,058	12,058	12,058
	No shifting	9012 (74.74)	8928 (74.04)	8833 (73.25)
	From PHC to Non-PHC	1740 (14.43)	1769 (14.67)	1786 (14.81)
	From Non-PHC to PHC	1306 (10.83)	1361 (11.29)	1439 (11.93)
Health status change		12,058	12,058	12,058
	No change	7984 (66.21)	7939 (65.84)	8009 (66.42)
	Become worse	1626 (13.49)	2340 (19.41)	1954 (16.21)
	Become better	2448 (20.30)	1779 (14.75)	2095 (17.37)

**Table 5:** Logistic regression analysis of predictors of PHC preference using balanced longitudinal data

Variables	Model 1: Univariate analysis		Model 2: Multivariate analysis	
	OR	95%CI	OR	95%CI
**Predisposing factors**				
Gender (Reference = Female)				
Male	1.009	(0.972, 1.048)		
Age (Reference = 16 ~ 25)				
26 ~ 35	0.775***	(0.676, 0.888)	1.050	(0.903, 1.221)
36 ~ 45	0.784***	(0.687, 0.895)	1.032	(0.891, 1.196)
46 ~ 55	0.785***	(0.689, 0.894)	1.115	(0.963, 1.290)
56 ~ 65	0.744***	(0.652, 0.848)	1.286***	(1.108, 1.491)
≥ 66	0.603***	(0.527, 0.690)	1.398***	(1.198, 1.632)
Education (Reference = Junior high school and below)				
High school or secondary	0.478***	(0.452, 0.505)	0.837***	(0.784, 0.895)
University or college and above	0.172***	(0.159, 0.186)	0.530***	(0.480, 0.585)
Ethnicity (Reference = Han)				
Minority	1.193***	(1.102, 1.291)	0.840***	(0.768, 0.918)
Marital status (Reference = Unmarried)				
Married	1.122***	(1.050, 1.199)	0.917**	(0.848, 0.991)
Household size (Reference = 1 ~ 2 person)				
3–4 person	1.253***	(1.190, 1.319)	1.270***	(1.191, 1.355)
≥ 5 person	1.850***	(1.756, 1.949)	1.559***	(1.460, 1.665)
**Enabling factors**				
Household income (Reference = < 30 thousand)				
30 ~ 50 thousand	0.731***	(0.693, 0.771)	0.881***	(0.830, 0.936)
50 ~ 70 thousand	0.562***	(0.529, 0.597)	0.797***	(0.742, 0.855)
70 ~ 90 thousand	0.481***	(0.447, 0.517)	0.770***	(0.706, 0.838)
≥ 90 thousand	0.392***	(0.371, 0.413)	0.653***	(0.611, 0.698)
Employment status (Reverence = Not currently working)				
Currently working	1.728***	(1.657, 1.803)	1.317***	(1.249, 1.390)
Hukou (Reference = Agricultural)				
Non-agricultural	0.196***	(0.188, 0.205)	0.676***	(0.622, 0.734)
Social basic medical insurance status (Reference = UEBMI)				
URBMI	1.431***	(1.313, 1.560)	1.344***	(1.226, 1.472)
NCMS	6.609***	(6.245, 6.994)	2.864***	(2.613, 3.140)
FMS	0.955	(0.837, 1.091)	0.976	(0.848, 1.122)
Geographic region (Reference = East)				
Central	1.171***	(1.119, 1.225)	1.043	(0.991, 1.098)
West	1.556***	(1.483, 1.632)	1.090***	(1.031, 1.152)
Living area (Reference = Rural)				
Urban	0.332***	(0.319, 0.345)	0.675***	(0.643, 0.710)
**Needs-based factors**				
Health status (Reference = Good)				
Fair	0.880**	(0.836, 0.926)	0.907***	(0.856, 0.962)
Poor	0.658***	(0.627, 0.691)	0.641***	(0.603, 0.681)
Chronic disease (Reference = No)				
Yes	0.501***	(0.479, 0.525)	0.657***	(0.621, 0.695)
Hospitalization (Reference = No)				
Yes	0.383***	(0.362, 0.404)	0.429***	(0.403, 0.457)
**Time factors**				
Year (Reference = 2012)				
2014	0.840***	(0.795, 0.888)	0.814***	(0.764, 0.867)
2016	0.720***	(0.682, 0.761)	0.700***	(0.657, 0.745)
2018	0.635***	(0.602, 0.671)	0.651***	(0.611, 0.694)
Constant			1.572***	(1.306, 1.892)
Pseudo R^2^			0.156	

Model 2 includes all significant variables through backward stepwise logistic analysis. The number of observations in Model 1 and Model 2 was 12,058, with each had 4 respondents during the four waves of survey, and the total respondents were *N* = 12,058 × 4 = 48,232

OR refers to odds ratios; 95% CI refers to 95% confidence intervals

The results of Model 2 were statistically significant, *χ*^2^ =9475.77, *p* < 0.001

***p < 0.01, **p < 0.05, *p < 0.10

**Table 6:** Multinomial logistic regression analysis of PHC preference shift on health status change

Variables	Preference shifting (Beta)		
	From PHC to Non-PHC vs. Non-shifting ^a^	From Non-PHC to PHC vs. Non-shifting ^b^	From PHC to Non-PHC vs. From Non-PHC to PHC ^c^
**Predisposing factors**			
Gender (Reference = Female)			
Male	0.064**	0.004	0.060
Age (Reference = 16 ~ 25)			
26 ~ 35	−0.183	0.034	−0.217
36 ~ 45	−0.260**	− 0.069	− 0.191
46 ~ 55	− 0.318***	− 0.007	− 0.312*
56 ~ 65	− 0.366***	− 0.017	− 0.349**
≥ 66	− 0.518***	− 0.041	−0.478***
Education (Reference = Junior high school and below)			
High school or secondary	0.006	0.067	−0.060
University/college or above	−0.055	−0.127	0.072
Ethnicity (Reference = Han)			
Minority	0.106*	0.100	0.006
Marital status (Reference = Unmarried)			
Married	0.123**	0.083	0.040
Household size (Reference = 1 ~ 2 person)			
3–4 person	−0.076*	−0.072	−0.003
≥ 5 person	−0.147***	−0.078	− 0.069
**Enabling factors**			
Household income (Reference = < 30 thousand)			
30 ~ 50 thousand	0.059	0.068	−0.009
50 ~ 70 thousand	0.077	0.047	0.030
70 ~ 90 thousand	0.034	0.094	−0.060
≥ 90 thousand	0.059	0.012	0.071
Employment status (Reverence = Not currently working)			
Currently working	−0.192***	−0.131***	− 0.061
Hukou (Reference = Agricultural)			
Non-agricultural	−0.185***	0.106	−0.291***
Social basic medical insurance status (Reference = UEBMI)			
URBMI	0.224***	0.134*	0.090
NCMS	−0.029	0.245***	−0.273**
FMS	−0.063	−0.011	− 0.052
Geographic region (Reference = East)			
Central	−0.068**	− 0.020	−0.048
West	−0.014	0.041	−0.055
Living area (Reference = Rural)			
Urban	0.119***	0.168***	−0.048
**Needs-based factors**			
Health status change (Reference = No change)			
**Become worse**	0.295***	−0.046	0.341***
**Become better**	−0.068	0.290***	−0.358***
Chronic disease (Reference = No)			
Yes	0.311***	−0.008	0.319***
Hospitalization (Reference = No)			
Yes	0.765***	−0.121**	0.886***
Constant	−1.471***	−2.143***	0.671***
Pseudo R^2^	0.017	0.017	0.017

^a^ This column took Non-shifting group as the reference group, and the results indicated the group of shifting from PHC to Non-PHC compared to the reference group

^b^ This column took Non-shifting group as the reference group, and the results indicated the group of shifting from Non-PHC to PHC compared to the reference group

^c^ This column took the group of shifting from Non-PHC to PHC as the reference group, and the results indicated the group of shifting from PHC to Non-PHC compared to reference group

The total respondents were *N* = 12,058 × 3 = 36,174

The results of multinomial logistic regression were statistically significant, *χ*^2^ =917.72, *p* < 0.001

***p < 0.01, **p < 0.05, *p < 0.10

## Results

### Characteristics of the sample

Table 1 reports the descriptive statistics for the variables in this study. Of the 12,058 individuals in 2012, 72.6% reported that they usually chose PHC services. The study sample was equally divided between males and females (51.7% vs. 48.3%). Individuals aged between 46 and 55 accounted for the largest proportion (25.1%). The overwhelming majority were of Han ethnicity (93.4%), married (91.6%), and had junior high school or below education (81.0%). About 40.4% of the individuals were from the eastern region of China, and more than half of the study sample lived in rural areas (59.5%). Most of the individuals were at work (61.1%), and 43.5% of the individuals reported that their household income in the last year was between 30,000 and 90,000 RMB. Table 1 also reports the primary analysis of Chi-square tests of independence, and the results indicated significant differences in PHC preference for each set of explanatory variables except for gender.

Table 1 reports that the proportion of respondents who chose PHC decreased from 72.6% in 2012 to 62.8% in 2018. This phenomenon was stable in the pooled data reported in Table A1 in Appendix A. Table 2 reports the changes in chronic disease and hospitalization from 2012 to 2018. The proportion of respondents with chronic disease or hospitalization has been significantly increased. The proportion of individuals hospitalized within the chronic disease group increased from 21.9% in 2012 to 35.7% in 2018 (Table 3). Table 4 reports the information of preference shifting and health status changes. The results indicated that the proportions of individuals’ preference shifting from PHC to non-PHC were more than the shifting from non-PHC to PHC in each wave.

### Logistic regression models

Table 5 reports the results of the logistic regression analysis. The univariate logistic regression analysis (Model 1, Table 5) suggested that all the potential factors, except for gender, were significantly associated with the usually choosing of PHC services. Married respondents, minority residents, respondents from central or west regions of China, rural residents, and those at work tended to report a higher likelihood of seeing a doctor in PHC institutions than the reference group. Educational level and household income were negatively related to the likelihood. The univariate logistic regression also indicated that respondents’ likelihood of choosing PHC service decreased over time as the year variables reporting a negative relationship with the likelihood (χ^2^ =300.99, *p* = 0.000).

Based on the univariate logistic model results, the multivariate logistic regression model was performed using a backward stepwise method (Model 2, Table5). Model 2 identified the predictors that significantly determined the PHC preference. We checked the collinearity of independent variables, and the values of VIF were from 1.05 to 4.06, with all values below the conventional threshold value of 10 [22]. It indicated no serious collinearity problems. The results indicated that all the three sets of variables, except for gender, were statistically significant determinants of the PHC service preference.

First, in predisposing factors, age, education, ethnicity, marital status, and household size were significantly associated with PHC preference. The likelihood of choosing PHC increased with age, and individuals aged 66 or above were 39.8% more likely to choose the PHC than individuals of 16 ~ 25 years old (OR, 1.398; 95% CI, 1.198–1.632). However, individuals with higher education levels had a lower odds of choosing PHC. Compared with the reference group, respondents with university/college or above education background were 47.0% less likely to choose PHC (OR, 0.530; 95% CI, 0.480–0.585). Individuals with minority ethnicity (OR, 0.840; 95% CI, 0.768–0.918) or married status (OR, 0.917; 95% CI, 0.848–0.991) were less likely to choose the PHC services. Household size was positively associated with the PHC preference. The results demonstrated that the respondents whose household members more than five were 55.9% more likely to choose PHC (OR, 1.559; 95% CI, 1.460–1.665).

Second, in enabling factors, we were able to find that all the variables were significantly associated with usual PHC service choice. Respondents from western regions (OR, 1.090; 95% CI, 1.031–1.152) had a higher propensity to choose PHC services, and urban residents (OR, 0.675; 95% CI, 0.643–0.710) or respondent with non-agricultural Hukou (OR, 0.676; 95% CI, 0.662–0.734) were less likely to choose them. Social basic medical insurance type was another factor determining the PHC preference, and the results indicated that respondents enrolled in NCMS (OR, 2.864; 95% CI, 2.613–3.140) or URBMI (OR, 1.344; 95% CI, 1.226–1.472) were, with respectively, 186.4% or 34.4%, more likely to choose PHC service than those in UEBMI. The likelihood of PHC service preference decreased with household income. Those whose household income was more than 90,000 RMB were 34.7% less likely to choose PHC institutions when they usually saw a doctor (OR, 0.653; 95% CI, 0.611–0.698). We also found that respondents at work had a higher likelihood of choosing PHC services (OR, 1.317; 95% CI, 1.249–1.390).

Third, in needs-based factors, all the three variables were negatively associated with PHC preference. Individuals with poor health status were 35.9% less likely to choose PHC services (OR, 0.641; 95% CI, 0.603–0.681), and those who had one or more chronic diseases were 34.3% less likely to choose them (OR 0.657; 95% CI, 0.621–0.695). Those individuals who needed better medical service chose the tertiary hospitals or specialty hospitals. Primary healthcare institutions are limited in inpatient services, so respondents who had a hospitalization in the past 12 months were 57.1% less likely to choose PHC services (OR, 0.429; 95% CI, 0.403–0.457).

We also conducted the univariate logistic models (Model 1 in Table A2, Appendix A) and the multivariate logistic model (Model 2 in Table A2, Appendix A) with the pooled longitudinal data to acquire robust results. The results were consistent with that of Model 2 in Table 5, except for a slight difference in the FMS group of social basic medical insurance status. It might be caused by the decrease in group sample size when using the balanced longitudinal data. FMS has been gradually phased out since 1998, and the number of individuals with FMS was tiny.

Table A3 (in Appendix A) reports the results of analysis stratified by geographical variables. All the results were consistent with Model 2 in Table 5, except for a slight difference in the age variable. The likelihood of PHC preference of respondents living in rural areas increased as their age grew while similar trend was not observed in urban areas. Respondents aged 66 and older were 88.5% more likely to choose the PHC in rural areas than reference group. On the contrary, elder respondents (age > = 56) in urban areas did not have a higher preference for PHC compared with the reference group.

Considering the study only included a small number of needs-based factors, we used panel data models to deal with possible unobserved heterogeneity of respondents. Table A4 (in Appendix A) reports the analysis results by using fixed and random effect models. The results indicated that the needs-based factors’ relationship with PHC preference was similar to Model 2 in Table 5.

### Trend in PHC preference: multinomial logistic regression

Model 2 in Table 5 also examined the time effect of PHC service preference. The results demonstrated that, over time, the likelihood of choosing PHC as the usual choice of care decreased from 2014. Compare to the 2012 group, the likelihood of PHC service preference decreased by 18.6% (OR, 0.814; 95% CI, 0.764–0.867) in 2014, 30.0% (OR, 0.700; 95% CI, 0.657–0.745) in 2016, and 34.9% (OR, 0.651; 95% CI, 0.611–0.694) in 2018. This effect was robust in Model 2 in Table A2 (Appendix A) when analysis was performed with pooled longitudinal data. These results indicated that when fixing other variables, an individual was less and less likely to use PHC services when seeing a doctor over time.

PHC institutions were limited in providing specialist medical services [23]. The decreasing likelihood of choosing PHC service might be related to the situation that more and more residents needed specialist medical services that were not provided by PHC institutions. It might be associated with residents’ health status. Thus, lagged health status variables were also introduced into the logistic regression models (Model 1 and 2 in Table A5, Appendix A). The results indicated that lagged health status also significantly associated with an individual’s PHC preference. Combined with the information in Table 4, we found that these shifting behaviors between PHC institutions and higher-level hospitals might be related to residents’ health status changes. Therefore, we performed multinomial logistic regressions to examine the relationship between the individuals’ shifting behavior and their health status changes (Table 6). In multinomial logistic models, the shifting behaviors were coded as the outcome variables, and different shifting behaviors were compared with each other.

The results in Table 6 demonstrated that the shifting behaviors were significantly associated with health status changes. In the group of shifting from PHC to Non-PHC (compared with individuals in the non-shifting group), those who became worse in health status were more likely to shift from PHC to non-PHC (*β*= 0.295, *p* < 0.01), and the likelihood of shifting increased 34.3% (*e*^0.295^ =1.343; OR, 1.343). On the contrary, in the group of shifting from non-PHC to PHC (compared with individuals in the non-shifting group), those who became better in health status were more likely to shift from non-PHC to PHC (*β*= 0.290, *p* < 0.01), and the likelihood of shifting increased 33.6% (*e*^0.290^ =1.336; OR, 1.336). This result was stable when the model was performed to compare the group of shifting from PHC to non-PHC with the group of shifting from non-PHC to PHC. Therefore, the decreasing trend in PHC service preference was highly associated with residents’ health status. Besides, the shifting behaviors and health status changing variables were also coded by comparing 2018 data with 2012 data. We conducted the multinomial logistic models again using the renewed variables (Table A6 in Appendix A), and the results were similar to those in Table 6.

## Discussion

This study examined the determinants of PHC service preference and the change of preference over time in China’s context. To the best of our knowledge, it is the first study that examined the trend of choosing PHC service by using a nationwide longitudinal data. We found that all the variables (except for gender) were statistically significant determinants of PHC preference. Notably, the residents’ likelihood of choosing PHC service decreased over time from 2012 to 2018.

With respect to the determinants of PHC preference, we found that residents with poor health status or chronic disease were less likely to choose the PHC services in China. This finding is inconsistent with the study of patients’ experience with PHC in Changchun in China, indicating that patients with poor health status or chronic disease were more likely to choose PHC [2]. The possible explanations for this finding might be that it was caused by the differences in study sample. Our sample was the nationally representative data with patients and non-patients, while the previous study was focused on the patients in PHC institutions in a regional area. Difference in study sample and disparity in PHC services quality between different areas impacted the residents’ choice of PHC services. We found that the regional differences in PHC preference were significant. It was highly associated with the imbalance of PHC service quality and resource allocation between different areas [24]. For example, rural PHC services in western China were weaker than that of eastern and central areas [25]. This gap was rooted in the inequality in health resources inputs in different areas and different levels of medical institutions. Studies on the PHC resource allocation in mainland China indicated that inequality in the geographical distribution of health resources was significant [26, 27]. PHC institutions in more than 80% of the provinces were inefficient, and the productivity of institutions declined from 2012 to 2016 [28]. Unbalanced medical resource distribution between PHC institutions and higher-tier hospitals led to an over-reliance on general and specialty hospitals [26, 29]. More health resources were allocated to hospitals, especially hospitals in the city of eastern areas, and the inequality between hospitals and PHC institutions in resource allocation indirectly impacted the patients’ evaluation of PHC services and their preference [27].

The decreasing trend in the likelihood of PHC preference might be associated with the population’s health status and the need for more specialist medical services in China. PHC was proved to provide healthcare at a lower cost and accepted by more and more residents [30–32]. High-quality PHC services were positively associated with the improvement in health outcomes and had no difference in providing services for patients with non-communicable diseases compared with higher-level hospitals [33, 34]. However, residents’ preference for PHC in China decreased between 2012 and 2018. One possible explanation might be that the services provided by PHC institutions cannot meet the patients’ needs well, and due to the health status changes, they have had to seek medical services provided by higher-level hospitals. Our study indicated that the prevalence of chronic diseases increased over time, and patients with chronic diseases having been hospitalized increased between 2012 and 2018. It was consistent with previous studies. For example, the prevalence of chronic obstructive pulmonary disease among people aged 40 years and over increased from 2.7% in 1990 to 13.6% in 2015 [35]. The numbers and rates per 100,000 population for stroke increased between 1990 and 2017, and pre-existing chronic diseases such as hypertension and diabetes mellitus were the main contributors [36]. A population of 1.7 million adults aged 35–75 years screening project indicated that the prevalence of hypertension increased in China, and 44.7% of the sample had hypertension [37].

The other explanation of the decreasing trend in PHC preference might be that limitation in PHC quality and the lack of referral system give residents the freedom to choose medical services at different levels. PHC institutions are the ideal place to treat and manage hypertension and diabetes. However, the quality of PHC may limit patients’ accessibility and divert patients to hospitals. Studies about service quality satisfaction and trust indicated that patients rated PHC institutions lower than higher-tier hospitals [14]. Besides, there is no referral system in China, and patients can directly access hospital care without a referral from PHC institutions [6, 9, 38, 39]. It is easy for patients to shift their primary care provider. As a result, the PHC system did not achieve its target of managing the growing needs of non-communicable diseases in China, and the congestion of patients in higher-tier hospitals was not released [40]. Evidence showed that policies benefiting patients and providers, such as better diabetes care and availability of medications, were related to improved quality of care [41]. China’s health sectors need to pay attention to this situation and adjust the medical services in PHC institutions in time. Additionally, enhancement of training quality for PHC physicians, establishing performance accountability, and strengthening the coordination between PHC institutions and hospitals were strongly recommended to improve the PHC quality in China [1].

There are several limitations associated with this study. First, we excluded respondents who did not complete the four surveys and those who had missing data, which limits the extrapolation of the results to its target population. Second, we did not include certain critical enabling or needs-based factors because they were absent from the CFPS. Due to the data incompleteness, we could not measure some essential factors such as accessibility to PHC and disease-related information. PHC preference was highly related to needs-based factors, and our study only considered a small number of determinants. Third, the respondents were the entire population but not the patients in China, and we only measured their usual preference in choosing PHC institutions, which might be biased. Preference for PHC institutions is different from PHC utilization because specific situations may impact an individual’s actual behavior in seeing a doctor, such as the type and severity of the diseases and characteristics of the medical institutions. Data from *China Health Statistical Yearbook* indicated that in the total medical service utilization, the proportion of PHC utilization decreased from 63.83% in 2009 to 54.11% in 2019. This also indirectly confirms our research findings. Finally, limited to the panel data used in this study, we can only present associations between determinant factors and PHC preference. It cannot verify the causal relationship between the shifting of PHC preference and health status change.

## Conclusion

This study contributes to our understanding of the trend and determinants of PHC service preference in China. The individuals’ socio-economic circumstances and health status factors determined their preference for PHC service. In particular, the likelihood of individuals’ preference for PHC services decreased over time. It was contrary to the objective of health reform in 2009 to improve the utilization of PHC. The study results might help policymakers understand the determinants of PHC service preference better and predict the future utilization trend. Policymakers may wish to adjust the service items in PHC facilities and strengthen the coordination of service providing between PHC institutions and tertiary hospitals. Capacities in PHC institutions should be improved to face the challenge of increasing demands of chronic disease.

## Supplementary Information

**Additional file 1: Table A1.** Descriptive information on PHC preference under Andersen Model using pooled data. **Table A2**. Logistic regression analysis of predictors of PHC preference using pooled data. **Table A3.** Determinants of PHC preference by geographical groups using balanced longitudinal data. **Table A4.** Logistic regression analysis of predictors of PHC preference using panel data models. **Table A5.** Logistic regression analysis of predictors of PHC preference using lagged health status variable. **Table A6.** Multinomial logistic regression analysis of PHC preference shift on health status change (2018 compare with 2012).

## Data Availability

The data that support the findings of this study are available from the Institute of Social Science Survey at the Peking University, and can be found on their website: http://www.isss.pku.edu.cn/cfps.
