# Supplementary material for: The trend in primary health care preference in China: a cohort study of 12,508 residents from 2012 to 2018
Source: BMC Health Serv Res. 2021 Aug 3;21:768. doi: 10.1186/s12913-021-06790-w (PMC8336283; doi:10.1186/s12913-021-06790-w)
Supplement: Supplementary file 1 — Additional file 1: Table A1. Descriptive information on PHC preference under Andersen Model using pooled data. Table A2. Logistic regression analysis of predictors of PHC preference using pooled data. Table A3. Determinants of PHC preference by geographical groups using balanced longitudinal data. Table A4. Logistic regression analysis of predictors of PHC preference using panel data models. Table A5. Logistic regression analysis of predictors of PHC preference using lagged health status variable. Table A6. Multinomial logistic regression analysis of PHC preference shift on health status change (2018 compare with 2012). [file 12913_2021_6790_MOESM1_ESM.docx]

**Appendix A**

**Table A1 Descriptive information on PHC preference under Andersen Model using pooled data**

| Factors | PHC N (%)  (n=63342) | Non-PHC N (%)  (n=36230) | Total N (%)  (n=99572) | $\chi^{2}$ | *P* |
| --- | --- | --- | --- | --- | --- |
| **Time factors** |  |  |  |  |  |
| Year |  |  |  | 668.228 | 0.000 |
| 2012 | 17497 (69.27) | 7762 (30.73) | 25259 (100) |  |  |
| 2014 | 16480 (64.93) | 8903 (35.07) | 25383 (100) |  |  |
| 2016 | 15415 (61.10) | 9816 (38.90) | 25231 (100) |  |  |
| 2018 | 13950 (58.86) | 9749 (41.14) | 23699 (100) |  |  |
| **Predisposing factors** |  |  |  |  |  |
| Gender |  |  |  | 10.852 | 0.001 |
| Female | 31858 (63.12) | 18615 (36.88) | 50473 (100) |  |  |
| Male | 31484 (64.12) | 17615 (35.88) | 49099 (100) |  |  |
| Age |  |  |  | 206.618 | 0.000 |
| 16~25 | 4285 (64.52) | 2356 (35.48) | 6641 (100) |  |  |
| 26~35 | 10340 (59.92) | 6916 (40.08) | 17256 (100) |  |  |
| 36~45 | 12358 (65.26) | 6580 (34.74) | 18938 (100) |  |  |
| 46~55 | 14934 (65.55) | 7848 (34.45) | 22782 (100) |  |  |
| 56~65 | 12259 (64.56) | 6730 (35.44) | 18989 (100) |  |  |
| ≥66 | 9166 (61.25) | 5800 (38.75) | 14966 (100) |  |  |
| Education |  |  |  | 5.8$\times$10^3^ | 0.000 |
| Junior high school and below | 53651 (69.37) | 23690 (30.63) | 77341 (100) |  |  |
| High school or secondary | 6710 (51.63) | 6287 (48.37) | 12997 (100) |  |  |
| University or college and above | 2981 (32.28) | 6253 (67.72) | 9234 (100) |  |  |
| Ethnicity |  |  |  | 14.915 | 0.000 |
| Han | 58038 (63.44) | 33448 (36.56) | 91486 (100) |  |  |
| Minority | 5304 (65.69) | 2782 (34.41) | 8086 (100) |  |  |
| Marital status |  |  |  | 47.704 | 0.000 |
| Unmarried | 8514 (61.01) | 5543 (38.99) | 13956 (100) |  |  |
| Married | 54828 (64.04) | 30788 (35.96) | 85616 (100) |  |  |
| Household size |  |  |  | 1.3$\times$10^3^ | 0.000 |
| 1~2 person | 10514 (56.02) | 8255 (43.98) | 18769 (100) |  |  |
| 3-4 person | 23133 (60.69) | 14985 (39.31) | 38118 (100) |  |  |
| ≥5 person | 29695 (69.57) | 12990 (30.43) | 42685 (100) |  |  |
| **Enabling factors** |  |  |  |  |  |
| Household income |  |  |  | 3.5$\times$10^3^ | 0.000 |
| <30 thousand | 28275 (72.38) | 10791 (27.62) | 39066 (100) |  |  |
| 30~50 thousand | 14138 (65.44) | 7468 (34.56) | 21606 (100) |  |  |
| 50~70 thousand | 7930 (59.84) | 5322 (40.16) | 13252 (100) |  |  |
| 70~90 thousand | 4717 (57.45) | 3494 (42.55) | 8211 (100) |  |  |
| ≥90 thousand | 8282 (47.50) | 9155 (52.50) | 17437 (100) |  |  |
| Employment status |  |  |  | 817.339 | 0.000 |
| Not currently working | 16732 (56.88) | 12683 (43.12) | 29415 (100) |  |  |
| Currently working | 46610 (44.44) | 23547 (33.56) | 70157 (100) |  |  |
| Hukou |  |  |  | 1.1$\times$10^4^ | 0.000 |
| Agricultural | 53628 (73.01) | 19823 (26.99) | 73451 (100) |  |  |
| Non-agricultural | 9714 (37.19) | 16407 (62.81) | 26121 (100) |  |  |
| Social basic medical insurance status |  |  |  | 1.3$\times$10^4^ | 0.000 |
| UEBMI | 4783 (32.12) | 10110 (67.88) | 14893 (100) |  |  |
| URBMI | 3495 (40.89) | 5053 (59.11) | 8548 (100) |  |  |
| NCMS | 54309 (73.82) | 19265 (26.18) | 73574 (100) |  |  |
| FMS | 755 (29.53) | 1802 (70.47) | 2557 (100) |  |  |
| Geographic region |  |  |  | 880.866 | 0.000 |
| East | 24062 (58.91) | 16781 (41.09) | 40843 (100) |  |  |
| Central | 18930 (63.96) | 10665 (36.04) | 29595 (100) |  |  |
| West | 20350 (69.85) | 8784 (30.15) | 29134 (100) |  |  |
| Living area |  |  |  | 6.6$\times$10^3^ | 0.000 |
| Rural | 39844 (75.19) | 13150 (24.81) | 52994 (100) |  |  |
| Urban | 23498 (50.45) | 23080 (49.55) | 46578 (100) |  |  |
| **Needs-based factors** |  |  |  |  |  |
| Health status |  |  |  | 288.463 | 0.000 |
| Good | 42251 (64.98) | 22768 (35.02) | 65019 (100) |  |  |
| Fair | 10818 (64.14) | 6048 (35.86) | 16866 (100) |  |  |
| Poor | 10273 (58.08) | 7414 (41.92) | 17687 (100) |  |  |
| Chronic disease |  |  |  | 1.3$\times$10^3^ | 0.000 |
| No | 54596 (66.12) | 27973 (33.88) | 82569 (100) |  |  |
| Yes | 8746 (51.44) | 8257 (48.56) | 17003 (100) |  |  |
| Hospitalization |  |  |  | 2.1$\times$10^3^ | 0.000 |
| No | 57833 (66.24) | 29470 (33.76) | 87303 (100) |  |  |
| Yes | 5509 (44.90) | 6760 (55.10) | 12269 (100) |  |  |

**Table A2 Logistic regression analysis of predictors of PHC preference using pooled data**

| Variables | Model 1: Univariate analysis | |  | Model 2: Multivariate analysis | |
| --- | --- | --- | --- | --- | --- |
|  | OR | 95%CI |  | OR | 95%CI |
| **Predisposing factors** |  |  |  |  |  |
| Gender (Reference = Female) |  |  |  |  |  |
| Male | 1.044*** | (1.017, 1.072) |  |  |  |
| Age (Reference = 16~25) |  |  |  |  |  |
| 26~35 | 0.822*** | (0.775, 0.872) |  | 1.067* | (0.998, 1.141) |
| 36~45 | 1.033 | (0.974, 1.095) |  | 1.181*** | (1.103, 1.265) |
| 46~55 | 1.046 | (0.988, 1.108) |  | 1.285*** | (1.200, 1.375) |
| 56~65 | 1.002 | (0.945, 1.062) |  | 1.477*** | (1.377, 1.585) |
| ≥66 | 0.869*** | (0.818, 0.923) |  | 1.642*** | (1.525, 1.767) |
| Education (Reference = Junior high school and below) |  |  |  |  |  |
| High school or secondary | 0.471*** | (0.454, 0.489) |  | 0.796*** | (0.762, 0.832) |
| University or college and above | 0.211*** | (0.201, 0.220) |  | 0.573*** | (0.540, 0.608) |
| Ethnicity (Reference = Han) |  |  |  |  |  |
| Minority | 1.098*** | (1.047, 1.153) |  | 0.712*** | (0.674, 0.752) |
| Marital status (Reference = Unmarried) |  |  |  |  |  |
| Married | 1.138*** | (1.097,1.181) |  | 0.958* | (0.916, 1.001) |
| Household size (Reference = 1~2 person) |  |  |  |  |  |
| 3-4 person | 1.212*** | (1.170, 1.256) |  | 1.221*** | (1.170, 1.275) |
| ≥5 person | 1.795*** | (1.732, 1.860) |  | 1.490*** | (1.426, 1.557) |
| **Enabling factors** |  |  |  |  |  |
| Household income (Reference = <30 thousand) |  |  |  |  |  |
| 30~50 thousand | 0.723*** | (0.697, 0.749) |  | 0.868*** | (0.834, 0.903) |
| 50~70 thousand | 0.569*** | (0.546, 0.593) |  | 0.784*** | (0.748, 0.822) |
| 70~90 thousand | 0.515*** | (0.491, 0.541) |  | 0.789*** | (0.746, 0.836) |
| ≥90 thousand | 0.345*** | (0.333, 0.358) |  | 0.609*** | (0.581, 0.638) |
| Employment status (Reverence = Not currently working) |  |  |  |  |  |
| Currently working | 1.500*** | (1.459, 1.543) |  | 1.347*** | (1.300, 1.395) |
| Hukou (Reference = Agricultural ) |  |  |  |  |  |
| Non-agricultural | 0.218*** | (0.212, 0.225) |  | 0.657*** | (0.624, 0.691) |
| Social medical insurance status (Reference = UEBMI) |  |  |  |  |  |
| URBMI | 1.462*** | (1.384, 1.545) |  | 1.362*** | (1.285, 1.444) |
| NCMS | 5.959*** | (5.736, 6.190) |  | 2.579*** | (2.435, 2.731) |
| FMS | 0.886*** | (0.808, 0.971) |  | 0.877*** | (0.797, 0.965) |
| Geographic region (Reference = East) |  |  |  |  |  |
| Central | 1.238*** | (1.200, 1.277) |  | 1.065*** | (1.028, 1.103) |
| West | 1.616*** | (1.565, 1.668) |  | 1.089*** | (1.049, 1.131) |
| Living area (Reference = Rural) |  |  |  |  |  |
| Urban | 0.336*** | (0.327, 0.345) |  | 0.673*** | (0.651, 0.696) |
| **Needs-based factors** |  |  |  |  |  |
| Health status (Reference = Good) |  |  |  |  |  |
| Fair | 0.964** | (0.930, 0.999) |  | 0.965* | (0.927, 1.005) |
| Poor | 0.747*** | (0.722, 0.772) |  | 0.691*** | (0.662, 0.721) |
| Chronic disease (Reference = No) |  |  |  |  |  |
| Yes | 0.543*** | (0.525, 0.561) |  | 0.662*** | (0.636, 0.689) |
| Hospitalization (Reference = No) |  |  |  |  |  |
| Yes | 0.415*** | (0.400, 0.431) |  | 0.439*** | (0.420, 0.459) |
| **Time factors** |  |  |  |  |  |
| Year (Reference = 2012) |  |  |  |  |  |
| 2014 | 0.821*** | (0.791, 0.852) |  | 0.801*** | (0.769, 0.836) |
| 2016 | 0.697*** | (0.671, 0.723) |  | 0.674*** | (0.647, 0.703) |
| 2018 | 0.635*** | (0.612, 0.659) |  | 0.654*** | (0.627, 0.683) |
| Constant |  |  |  | 1.317*** | (1.194, 1.453) |
| Pseudo R^2^ |  |  |  | 0.149 |  |

Model 2 includes all significant variables through backward stepwise logistic analysis. The number of respondents in Model 1 and Model 2 was 99572, including all the four waves of observations.

OR refers to odds ratios; 95% CI refers to 95% confidence intervals.

The results of Model 2 were statistically significant, $\chi^{2}$= 19423.50, p<0.001.

****p*<0.01, ***p*<0.05, **p*<0.10.

**Table A3 Determinants of PHC preference by geographical groups using balanced longitudinal data**

| Variables | Geographical region (OR) | | |  | Living area (OR) | |
| --- | --- | --- | --- | --- | --- | --- |
|  | East | Central | West |  | Rural | Urban |
| **Predisposing factors** |  |  |  |  |  |  |
| Age (Reference = 16~25) |  |  |  |  |  |  |
| 26~35 | 0.930 | 1.219 | 1.076 |  | 1.322*** | 0.814* |
| 36~45 | 0.935 | 1.116 | 1.111 |  | 1.385*** | 0.761** |
| 46~55 | 1.089 | 1.056 | 1.211 |  | 1.506*** | 0.809* |
| 56~65 | 1.233* | 1.182 | 1.421** |  | 1.586*** | 1.025 |
| ≥66 | 1.223 | 1.444* | 1.480*** |  | 1.885*** | 0.991 |
| Education (Reference = Junior high school and below) |  |  |  |  |  |  |
| High school or secondary | 0.853*** | 0.833*** | 0.777*** |  | 0.918 | 0.788*** |
| University or college and above | 0.475*** | 0.590*** | 0.509*** |  | 0.612*** | 0.510*** |
| Ethnicity (Reference = Han) |  |  |  |  |  |  |
| Minority | 0.768*** | 1.222 | 0.868** |  | 0.863*** | 0.835** |
| Marital status (Reference = Unmarried) |  |  |  |  |  |  |
| Married | 0.954 | 0.865* | 0.863* |  | 0.798*** | 1.038 |
| Household size (Reference = 1~2 person) |  |  |  |  |  |  |
| 3-4 person | 1.289*** | 1.441*** | 1.058 |  | 1.267*** | 1.288*** |
| ≥5 person | 1.684*** | 1.791*** | 1.217*** |  | 1.549*** | 1.575*** |
| **Enabling factors** |  |  |  |  |  |  |
| Household income (Reference = <30 thousand) |  |  |  |  |  |  |
| 30~50 thousand | 0.821*** | 0.836*** | 0.980 |  | 0.904** | 0.817*** |
| 50~70 thousand | 0.729*** | 0.829*** | 0.804*** |  | 0.870*** | 0.702*** |
| 70~90 thousand | 0.631*** | 0.881 | 0.811** |  | 0.776*** | 0.711*** |
| ≥90 thousand | 0.604*** | 0.654*** | 0.649*** |  | 0.687*** | 0.586*** |
| Employment status (Reverence = Not currently working) |  |  |  |  |  |  |
| Currently working | 1.232*** | 1.356*** | 1.447*** |  | 1.339*** | 1.322*** |
| Hukou (Reference = Agricultural ) |  |  |  |  |  |  |
| Non-agricultural | 0.707*** | 0.543*** | 0.789** |  | 0.778*** | 0.671*** |
| Social medical insurance status (Reference = UEBMI) |  |  |  |  |  |  |
| URBMI | 1.518*** | 1.231** | 1.079 |  | 1.006 | 1.386*** |
| NCMS | 3.174*** | 2.312*** | 2.374*** |  | 2.592*** | 2.805*** |
| FMS | 1.150 | 0.945 | 0.533*** |  | 0.953 | 0.981 |
| Geographic region (Reference = East) |  |  |  |  |  |  |
| Central |  |  |  |  | 1.136*** | 0.955 |
| West |  |  |  |  | 0.951 | 1.346*** |
| Living area (Reference = Rural) |  |  |  |  |  |  |
| Urban | 0.666*** | 0.541*** | 0.881*** |  |  |  |
| **Needs-based factors** |  |  |  |  |  |  |
| Health status (Reference = Good) |  |  |  |  |  |  |
| Fair | 0.915* | 0.879** | 0.893** |  | 0.851*** | 0.949 |
| Poor | 0.682*** | 0.554*** | 0.690*** |  | 0.620*** | 0.678*** |
| Chronic disease (Reference = No) |  |  |  |  |  |  |
| Yes | 0.689*** | 0.665*** | 0.615*** |  | 0.637*** | 0.689*** |
| Hospitalization (Reference = No) |  |  |  |  |  |  |
| Yes | 0.404*** | 0.495*** | 0.405*** |  | 0.421*** | 0.443*** |
| **Time factors** |  |  |  |  |  |  |
| Year (Reference = 2012) |  |  |  |  |  |  |
| 2014 | 0.903** | 0.768*** | 0.725*** |  | 0.786*** | 0.839*** |
| 2016 | 0.748*** | 0.666*** | 0.652*** |  | 0.694*** | 0.707*** |
| 2018 | 0.673*** | 0.655*** | 0.603*** |  | 0.573*** | 0.747*** |
| Constant | 1.515*** | 2.174*** | 2.110*** |  | 1.573*** | 1.253* |
| N | 19605 | 14916 | 13711 |  | 27251 | 20981 |
| Chi-square | 4410.97 | 3336.66 | 1652.75 |  | 2024.29 | 4534.47 |
| Pseudo R^2^ | 0.172 | 0.177 | 0.104 |  | 0.070 | 0.157 |

OR refers to odd ratios. ****p*<0.01, ***p*<0.05, **p*<0.10.

**Table A4 Logistic regression analysis of predictors of PHC preference using panel data models**

| Variables | Model 1: Fixed effect | |  | Model 2: Random effect | |
| --- | --- | --- | --- | --- | --- |
|  | Beta | 95%CI |  | Beta | 95%CI |
| **Predisposing factors** |  |  |  |  |  |
| Gender (Reference = Female) |  |  |  |  |  |
| Male | -0.860 | (-2.165, 0.446) |  | 0.028 | (-0.048, 0.105) |
| Age (Reference = 16~25) |  |  |  |  |  |
| 26~35 | -0.424*** | (-0.677, -0.172) |  | -0.140 | (-0.345, 0.065) |
| 36~45 | -0.950*** | (-1.271, -0.630) |  | -0.200* | (-0.412, 0.012) |
| 46~55 | -1.256*** | (-1.605, -0.907) |  | -0.194* | (-0.405, 0.016) |
| 56~65 | -1.532*** | (-1.915, -1.150) |  | -0.078 | (-0.293, 0.135) |
| ≥66 | -1.869*** | (-2.285, -1.453) |  | -0.121 | (-0.344, 0.102) |
| Education (Reference = Junior high school and below) |  |  |  |  |  |
| High school or secondary | -0.295 | (-0.792, 0.201) |  | -0.329*** | (-0.445, -0.213) |
| University or college and above | -0.734** | (-1.409, -0.058) |  | -1.037*** | (-1.209, -0.865) |
| Ethnicity (Reference = Han) |  |  |  |  |  |
| Minority |  |  |  | -0.263*** | (-0.418, -0.107) |
| Marital status (Reference = Unmarried) |  |  |  |  |  |
| Married | 0.053 | (-0.157, 0.264) |  | -0.062 | (-0.185, 0.061) |
| Household size (Reference = 1~2 person) |  |  |  |  |  |
| 3-4 person | 0.176*** | (0.047, 0.305) |  | 0.295*** | (0.200, 0.389) |
| ≥5 person | 0.239*** | (0.093, 0.384) |  | 0.509*** | (0.407, 0.605) |
| **Enabling factors** |  |  |  |  |  |
| Household income (Reference = <30 thousand) |  |  |  |  |  |
| 30~50 thousand | -0.074* | (-0.160, 0.012) |  | -0.156*** | (-0.233, -0.078) |
| 50~70 thousand | -0.110** | (-0.215, -0.005) |  | -0.264*** | (-0.356, -0.172) |
| 70~90 thousand | -0.072 | (-0.201, -0.057) |  | -0.274*** | (-0.386, -0.162) |
| ≥90 thousand | -0.218*** | (-0.328, -0.109) |  | -0.495*** | (-0.585, -0.404) |
| Employment status (Reverence = Not currently working) |  |  |  |  |  |
| Currently working | 0.036 | (-0.050, 0.122) |  | 0.166*** | (0.095, 0.238) |
| Hukou (Reference = Agricultural ) |  |  |  |  |  |
| Non-agricultural | -0.056 | (-0.281, 0.168) |  | -0.572*** | (-0.700, -0.445) |
| Social basic medical insurance status (Reference = UEBMI) |  |  |  |  |  |
| URBMI | 0.013 | (-0.161, 0.187) |  | 0.301*** | (0.168, 0.434) |
| NCMS | 0.409*** | (0.204, 0.614) |  | 1.256*** | (1.116, 1.396) |
| FMS | -0.020 | (-0.251, 0.210) |  | 0.007 | (-0.184, 0.198) |
| Geographic region (Reference = East) |  |  |  |  |  |
| Central | -0.133 | (-0.753, 0.487) |  | 0.070 | (-0.020, 0.160) |
| West | 0.789** | (0.057, 1.521) |  | 0.129*** | (0.033, 0.225) |
| Living area (Reference = Rural) |  |  |  |  |  |
| Urban | -0.698*** | (-0.880, -0.517) |  | -0.618*** | (0.700, 0.536) |
| **Needs-based factors** |  |  |  |  |  |
| Health status (Reference = Good) |  |  |  |  |  |
| Fair | -0.146*** | (-0.233, -0.058) |  | -0.124*** | (-0.201, -0.048) |
| Poor | -0.477*** | (-0.580, -0.375) |  | -0.543*** | (-0.627, -0.459) |
| Chronic disease (Reference = No) |  |  |  |  |  |
| Yes | -0.505*** | (-0.587, -0.423) |  | -0.569*** | (-0.643, -0.496) |
| Hospitalization (Reference = No) |  |  |  |  |  |
| Yes | -0.868*** | (-0.957, -0.779) |  | -1.069*** | (-1.151, -0.986) |

The results of Model 1 were statistically significant, $\chi^{2}$=1099.39, p<0.001.

The results of Model 2 were statistically significant, $\chi^{2}$=4363.39, p<0.001.

****p*<0.01, ***p*<0.05, **p*<0.10.

**Table A5 Logistic regression analysis of predictors of PHC preference using lagged health status variable**

| Variables | Model 1: Multivariate analysis | |  | Model 2: Multivariate analysis | |
| --- | --- | --- | --- | --- | --- |
|  | OR | 95%CI |  | OR | 95%CI |
| **Predisposing factors** |  |  |  |  |  |
| Age (Reference = 16~25) |  |  |  |  |  |
| 26~35 | 1.119 | (0.909, 1.378) |  | 1.109 | (0.901, 1.365) |
| 36~45 | 1.094 | (0.892, 1.343) |  | 1.073 | (0.874, 1.316) |
| 46~55 | 1.220* | (0.996, 1.496) |  | 1.186* | (0.968, 1.453) |
| 56~65 | 1.411*** | (1.149, 1.732) |  | 1.369*** | (1.115, 1.680) |
| ≥66 | 1.615*** | (1.308, 1.992) |  | 1.576*** | (1.278, 1.944) |
| Education (Reference = Junior high school and below) |  |  |  |  |  |
| High school or secondary | 0.841*** | (0.779, 0.907) |  | 0.847*** | (0.785, 0.914) |
| University or college and above | 0.515*** | (0.459, 0.577) |  | 0.513*** | (0.458, 0.575) |
| Ethnicity (Reference = Han) |  |  |  |  |  |
| Minority | 0.850*** | (0.767, 0.941) |  | 0.848*** | (0.765, 0.939) |
| Marital status (Reference = Unmarried) |  |  |  |  |  |
| Married | 0.877*** | (0.803, 0.958) |  | 0.880*** | (0.806, 0.962) |
| Household size (Reference = 1~2 person) |  |  |  |  |  |
| 3-4 person | 1.294*** | (1.203, 1.392) |  | 1.292*** | (1.202, 1.389) |
| ≥5 person | 1.585*** | (1.472, 1.706) |  | 1.579*** | (1.467, 1.700) |
| **Enabling factors** |  |  |  |  |  |
| Household income (Reference = <30 thousand) |  |  |  |  |  |
| 30~50 thousand | 0.894*** | (0.834, 0.958) |  | 0.906*** | (0.846, 0.970) |
| 50~70 thousand | 0.788*** | (0.726, 0.856) |  | 0.800*** | (0.737, 0.868) |
| 70~90 thousand | 0.747*** | (0.677, 0.825) |  | 0.762*** | (0.690, 0.841) |
| ≥90 thousand | 0.646*** | (0.598, 0.697) |  | 0.657*** | (0.609, 0.709) |
| Employment status (Reverence = Not currently working) |  |  |  |  |  |
| Currently working | 1.462*** | (1.370, 1.560) |  | 1.496*** | (1.403, 1.596) |
| Hukou (Reference = Agricultural ) |  |  |  |  |  |
| Non-agricultural | 0.701*** | (0.637, 0.770) |  | 0.707*** | (0.644, 0.777) |
| Social basic medical insurance status (Reference = UEBMI) |  |  |  |  |  |
| URBMI | 1.347*** | (1.213, 1.496) |  | 1.341*** | (1.208, 1.490) |
| NCMS | 2.897*** | (2.608, 3.218) |  | 20879*** | (2.592, 3.198) |
| FMS | 1.033 | (0.870, 1.226) |  | 1.040 | (0.876, 1.235) |
| Geographic region (Reference = East) |  |  |  |  |  |
| Central | 1.030 | (0.971, 1.092) |  | 1.032 | (0.973, 1.094) |
| West | 1.065* | (1.000, 1.135) |  | 1.065* | (0.999, 1.135) |
| Living area (Reference = Rural) |  |  |  |  |  |
| Urban | 0.693*** | (0.655, 0.733) |  | 0.697*** | (0.659, 0.737) |
| **Needs-based factors** |  |  |  |  |  |
| Health status (Reference = Good) |  |  |  |  |  |
| Fair | 0.928** | (0.866, 0.995) |  |  |  |
| Poor | 0.721*** | (0.667, 0.778) |  |  |  |
| Lagged health status (Reference = Good) |  |  |  |  |  |
| Fair | 1.026 | (0.960, 1.097) |  | 0.993 | (0.930, 1.060) |
| Poor | 0.909** | (0.843, 0.980) |  | 0.799*** | (0.746, 0.855) |
| Chronic disease (Reference = No) |  |  |  |  |  |
| Yes | 0.658*** | (0.617, 0.702) |  | 0.622*** | (0.584, 0.662) |
| Hospitalization (Reference = No) |  |  |  |  |  |
| Yes | 0.383*** | (0.357, 0.411) |  | 0.366*** | (0.341, 0.392) |
| **Time factors** |  |  |  |  |  |
| Year (Reference = 2014) |  |  |  |  |  |
| 2016 | 0.857*** | (0.806, 0.911) |  | 0.850*** | (0.801, 0.904) |
| 2018 | 0.796*** | (0.749, 0.846) |  | 0.790*** | (0.743, 0.839) |
| Constant | 1.097 | (0.861, 1.397) |  | 1.066 | (0.837, 1.357) |
| Pseudo R^2^ | 0.159 |  |  | 0.157 |  |

The number of observations in Model 1 and Model 2 was 12058, with each had 3 respondents during 2014-2018 waves of survey, and the total respondents were N=12058×3=36174.

OR refers to odds ratios; 95% CI refers to 95% confidence intervals.

The results of Model 1 were statistically significant, $\chi^{2}$=7375.14, p<0.001.

The results of Model 2 were statistically significant, $\chi^{2}$=7305.60, p<0.001.

****p*<0.01, ***p*<0.05, **p*<0.10.

**Table A6 Multinomial logistic regression analysis of PHC preference shift on health status change (2018 compare with 2012)**

| Variables | Preference shifting (Beta) | | |
| --- | --- | --- | --- |
|  | From PHC to Non-PHC vs. Non-shifting ^a^ | From Non-PHC to PHC vs. Non-shifting ^b^ | From PHC to Non-PHC vs. From Non-PHC to PHC ^c^ |
| **Predisposing factors** |  |  |  |
| Gender (Reference = Female) |  |  |  |
| Male | 0.077 | -0.015 | 0.092 |
| Age (Reference = 16~25) |  |  |  |
| 26~35 | -0.477 | 0.590 | -1.067* |
| 36~45 | -0.429 | 0.571 | -1.000 |
| 46~55 | -0.549* | 0.536 | -1.084* |
| 56~65 | -0.563* | 0.710 | -1.273** |
| ≥66 | -0.681** | 0.705 | -1.385** |
| Education (Reference = Junior high school and below) |  |  |  |
| High school or secondary | -0.030 | 0.092 | -0.122 |
| University/college or above | 0.211* | -0.082 | 0.293* |
| Ethnicity (Reference = Han) |  |  |  |
| Minority | 0.126 | 0.153 | -0.027 |
| Marital status (Reference = Unmarried) |  |  |  |
| Married | 0.147* | 0.061 | 0.086 |
| Household size (Reference = 1~2 person) |  |  |  |
| 3-4 person | -0.145** | -0.046 | -0.099 |
| ≥5 person | -0.301*** | -0.136 | -0.165 |
| **Enabling factors** |  |  |  |
| Household income (Reference = <30 thousand) |  |  |  |
| 30~50 thousand | 0.087 | 0.072 | 0.015 |
| 50~70 thousand | 0.224*** | 0.096 | 0.128 |
| 70~90 thousand | 0.007 | -0.041 | 0.048 |
| ≥90 thousand | 0.217*** | 0.085 | 0.132 |
| Employment status (Reverence = Not currently working) |  |  |  |
| Currently working | -0.315*** | 0.064 | -0.379*** |
| Hukou (Reference = Agricultural ) |  |  |  |
| Non-agricultural | -0.409*** | 0.224* | -0.632*** |
| Social basic medical insurance status (Reference = UEBMI) |  |  |  |
| URBMI | 0.252** | 0.011 | 0.241 |
| NCMS | -0.248** | 0.181 | -0.429*** |
| FMS | -0.132 | 0.041 | -0.173 |
| Geographic region (Reference = East) |  |  |  |
| Central | -0.077 | -0.031 | -0.046 |
| West | 0.009 | 0.041 | -0.031 |
| Living area (Reference = Rural) |  |  |  |
| Urban | 0.007 | 0.098 | -0.091 |
| **Needs-based factors** |  |  |  |
| Health status change (Reference = No change) |  |  |  |
| **Become worse** | **0.234***** | **-0.246**** | **0.480***** |
| **Become better** | -0.039 | **0.318***** | **-0.357***** |
| Chronic disease (Reference = No) |  |  |  |
| Yes | 0.236*** | -0.011 | 0.225** |
| Hospitalization (Reference = No) |  |  |  |
| Yes | 0.785*** | -0.179* | 0.964*** |
| Constant | -0.586* | -2.955*** | 2.369*** |
| Pseudo R^2^ | 0.024 | 0.024 | 0.024 |

^a^ This column took Non-shifting group as the reference group, and the results indicated the group of shifting from PHC to Non-PHC compared to the reference group.

^b^ This column took Non-shifting group as the reference group, and the results indicated the group of shifting from Non-PHC to PHC compared to the reference group.

^c^ This column took the group of shifting from Non-PHC to PHC as the reference group, and the results indicated the group of shifting from PHC to Non-PHC compared to reference group.

The total respondents were N=12058.

The results of multinomial logistic regression were statistically significant, $\chi^{2}$=458.32, p<0.001.

****p*<0.01, ***p*<0.05, **p*<0.10.
